# Diseases of the Abdomen

**Published:** 1902-12-20

**Authors:** 


*(To be Continued.)*

### Diseases of the Abdomen.

The treatment of gastric ulcer according to Robin [1] consists in at first complete rest in bed, a hot compress to the epigastrium, and nothing by the mouth; 4 enemata daily, consisting of 2 eggs, ʒiii. of peptone, 30 gr. of salt, ℥iii. of glucose (20 per cent.), and a few drops of tinct. opii. making ℥vi. altogether. Two other enemata of warm water are given daily. It is recommended by Fournier and Gros that this treatment should be continued for two weeks or even a month, but Robin has never found it necessary to prolong it longer than 12 days at most; frequently three or four days being sufficient. When the distressing symptoms have disappeared, then milk should be given by the mouth, the amount being gradually increased till four quarts are reached per diem. This must be maintained for not less than six months, when a mixed diet can be ordered for two weeks. At this time milk can be absolutely abandoned and the patient allowed meat, fish, eggs, vegetables and cooked fruits. Supplementary tonic treatment may also be necessary, and for this the subcutaneous injections of cacadylate of sodium is the most advantageous. It should be given in 1 grain doses for eight consecutive days, and repeated after eight days' rest until 40 days are reached.

Foulerton [2] describes a case of acute membranous gastritis due to pneumonic infection. The patient, a male aged 26, showed no indications during life of gastric disturbance. The wall of the stomach showed a new membranous formation which terminated abruptly at the pyloric and cardiac ends, and presented an appearance of acute poisoning with a mineral acid. There were hæmorrhages in various parts of the body, one the size of a shilling in the right lung. In culture the bacilli found were the diplococcus pneumoniæ, and the *B. mesentericus vulgatus.* In the stomach there were two kinds of bacteria found, chiefly the diplococcus pneumoniæ. The suggestion was that infection had taken place *viâ* the right tonsil, and the parasites had been swallowed and had set up a general invasion from the stomach.

Two cases are recorded of fatal tetany ensuing upon dilated conditions of the stomach. Calwell's case [3] was that of a young man aged 26, who had had gastric trouble for three years. He had recently lost weight considerably, and on January 11th, 1902, presented all the symptoms of dilated stomach. This organ was washed out on January 15th, and a test breakfast given. From the 16th he became progressively weaker. His hands were noticed to be in the "accoucheur" form, wrists and elbows partly flexed; toes and feet dorsiflexed. There were twitchings and spasms of the muscles generally, and increasing cardiac weakness ended the case, in spite of strychnia and other restoratives. The writer points out that this is now regarded as a clinical entity, that it is very fatal, the fatal cases numbering 75 per cent. either through delirium, rapid rise of temperature, or simple heart failure. Tetanoid convulsions are always fatal.

Soltau Fenwick's [4] case was also a man, and aged 64. He had a dilated stomach, which was washed out with warm water, pending a discussion on the advisability of surgical interference. On the next day he felt better, but at two o'clock he was seized with violent vomiting, which continued till 7 P.M., when tetany ensued. This affected all the limbs, but more especially the left. The forearms were flexed, pronated, and adducted, wrists bent, thumbs pressed into the palms of the hands, with fingers over them. Heels drawn upwards, soles inwards, slight trismus. This condition lasted for 16 hours, with very feeble pulse, and death resulted from cardiac failure. The writer states that death in the first attack of tetany is very rare. Trismus is always a sign of evil augury. It is possible that the passage of the tube, or the severe retching injured the hitherto-intact columnar epithelium, and allowed a poison to be absorbed.

[1] Amer. Jour. Med. Sci., March. [2] Lancet, April 12. [3] Brit. Med. Jour., June 28. [4] Ibid., March 8.

*(To be continued.)*

